# *accD* nuclear transfer of *Platycodon grandiflorum* and the plastid of early Campanulaceae

**DOI:** 10.1186/s12864-017-4014-x

**Published:** 2017-08-11

**Authors:** Chang Pyo Hong, Jihye Park, Yi Lee, Minjee Lee, Sin Gi Park, Yurry Uhm, Jungho Lee, Chang-Kug Kim

**Affiliations:** 1 Bioinformatics Team, Theragen Etex Bio Institute, Suwon, 443-270 South Korea; 2 Green Plant Institute, B-301, Heungdeok IT Valley, Giheung-gu, Yongin, 446-908 South Korea; 3 Department of Industrial Plant Science and Technology, Chungbuk National University, Cheongju, 362-763 South Korea; 4 Herbal Crop Research Division, National Institute of Horticultural and Herbal Science (NIHH), RDA, Eumseong, 369-873 South Korea; 5 Genomics Division, National Institute of Agricultural Science (NAS), RDA, Jeonju, 560-500 South Korea

**Keywords:** *accD*, Campanulaceae, Genomic rearrangement, Intron loss, Nuclear gene transfer, Plastid

## Abstract

**Background:**

Campanulaceae species are known to have highly rearranged plastid genomes lacking the acetyl-CoA carboxylase (ACC) subunit D gene (*accD*), and instead have a nuclear (nr)-*accD*. Plastid genome information has been thought to depend on studies concerning *Trachelium caeruleum* and genome announcements for *Adenophora remotiflora*, *Campanula takesimana,* and *Hanabusaya asiatica*. RNA editing information for plastid genes is currently unavailable for Campanulaceae. To understand plastid genome evolution in Campanulaceae, we have sequenced and characterized the chloroplast (cp) genome and nr-*accD* of *Platycodon grandiflorum*, a basal member of Campanulaceae.

**Results:**

We sequenced the 171,818 bp cp genome containing a 79,061 bp large single-copy (LSC) region, a 42,433 bp inverted repeat (IR) and a 7840 bp small single-copy (SSC) region, which represents the cp genome with the largest IR among species of Campanulaceae. The genome contains 110 genes and 18 introns, comprising 77 protein-coding genes, four RNA genes, 29 tRNA genes, 17 group II introns, and one group I intron. RNA editing of genes was detected in 18 sites of 14 protein-coding genes. *Platycodon* has an IR containing a 3′ *rps12* operon, which occurs in the middle of the LSC region in four other species of Campanulaceae (*T. caeruleum*, *A. remotiflora*, *C. takesimana,* and *H. asiatica*), but lacks *accD, clpP, infA,* and *rpl23,* as has been found in these four species*. Platycodon* nr-*accD* contains about 3.2 kb intron between nr-*accD.e1* and nr-*accD.e2* at the same insertion point as in other Campanulaceae. The phylogenies of the plastid genomes and *accD* show that *Platycodon* is basal in the Campanulaceae clade, indicating that IR disruption in Campanulaceae occurred after the loss of *accD, clpP, infA,* and *rpl23* in the cp genome, which occurred during plastid evolution in Campanulaceae.

**Conclusions:**

The plastid genome of *P. grandiflorum* lacks the rearrangement of the IR found in *T. caeruleum*, *A. remotiflora*, *C. takesimana,* and *H. asiatica*. The absence of *accD, clpP, infA,* and *rpl23* in the plastid genome is a synapomorphic characteristic of Campanulaceae. The chloroplast genome phylogeny supports the hypothesis that chloroplast genomic arrangement occurred after *accD* nuclear transfer and loss of the four genes in the plastid of early Campanulaceae as a lineage of asterids.

**Electronic supplementary material:**

The online version of this article (doi:10.1186/s12864-017-4014-x) contains supplementary material, which is available to authorized users.

## Background

Plastid organization is highly conserved among angiosperms. Most angiosperm plastids have a quadripartite structure with two copies of a large inverted repeat (IR) separated by small (SSC) and large (LSC) single-copy regions. The two copies of the IR facilitate flip–flop recombination, resulting in the presence of isoforms that differ in the orientation of the single copy regions. The early electron microscopic comparisons revealed that plastid genomes are circular in either monomeric or multimeric forms [1]. Substantial recent evidence suggests that the plastid genome has a more complex structure, with circular, linear, branched, and multimeric configurations that vary during plastid development [2–7].

Inverted repeat expansion and contraction occur in the plastid genome of land plants via a boundary shift of the border regions of IR/LSC and IR/SSC. In addition to these IR boundary shifts, there are a few cases where the IR has been severely reduced or even eliminated [8–13]. The major shift of the IR in the Campanulaceae species *Hanabusaya* and *Trachelium* provides examples of SC-to-IR transitions for six genes (*ycf1*, *rps15*, *ndhH*, *ndhA*, *ndhI*, and *ndhG*), with the exception of the 3′ *rps12* operon and IR-to-SC transitions for five of the six ancestral IR genes [13].

The majority of plastid genes are contained in operons and transcribed as poly-cistronic units, a feature that originated from a cyanobacterial ancestor. In angiosperm plastids, disruption of operons has been documented in three angiosperm families: Campanulaceae [14–16], Geraniaceae [10, 17], and Fabaceae [18–21]. In Campanulaceae, the plastomes have two disrupted operons: the *rps2* operon (*rps2* - *atpI* - *atpH* - *atpF* - *atpA*) and the *clpP* operon (*clpP* - 5′ *rps12* - *rpl20*) [15, 16]. In both cases, the relocated segments of the operons must have acquired new promoters to be transcribed; however, experimental studies have not been performed to determine how these segments are transcribed in their new locations [22].

The nature of the IR reduces the substitution rate. Zhu et al. [13] demonstrated that synonymous substitution rates are, on average, 3.7 times slower in IR genes than in SC genes, and that genes moved from the SC into the IR exhibit lower synonymous rates consistent with other IR genes, whereas genes moved from the IR into the SC exhibit higher rates consistent with other SC genes; the exceptions being in *Pelargonium* (Geraniaceae), *Plantago* (Plataginaceae), and *Silene* (Caryophyllaceae). In this paper, they used the comparison of the species *Hanabusaya* and *Trachelium* of Campanulaceae as the most illustrative single example of the effect of IR duplication on substitution rates.

Although many gene losses have been documented in angiosperms [23, 24], only a few of these events have been rigorously investigated [22]. It is widely known that plastid DNA is transferred to the nucleus at a high rate [25–27]; however, only a few functional gene transfers to the nucleus have been characterized in angiosperms.

The acetyl-CoA carboxylase (ACC) subunit D gene (*accD*) is known to be essential for leaf development in angiosperms [28]. The *accD* gene has been lost at least in seven times in angiosperm plastid genomes including Poales [29–31], Acoraceae [32, 33], Geraniaceae [34], Fabaceae [24], Campanulaceae [33], Oleaceae [35], and Rafflesiaceae [36]. In *Trifolium*, one copy of this gene was found in the nucleus [24]. In grasses, the prokaryotic multisubunit enzyme has been replaced by a plastid-targeted eukaryotic ACC [37, 38].

Campanulaceae, including Lobeliaceae (sensu APG III 2009), have experienced a high degree of gene order change. Although only one plastome sequence, that of *Trachelium caeruleum* [16], has been published, draft genomes have been completed for several other genera [15], and restriction site and gene maps have been published for many others [39, 40]. The most extensive comparisons have included gene maps for 18 genera of Campanulaceae [22, 39], where the authors estimated that the changes in gene order were due to a minimum of 42 inversions, 18 large insertions (>5 kb) of unknown origin, five IR expansions and contractions, and several putative transpositions [22]. The complete genome sequence of *Trachelium* [16], the least rearranged taxon examined by Cosner et al. [39], confirmed that at least seven inversions are present in this genome, but it did not provide any evidence of transposition as a mechanism underlying the observed changes in gene order [22].

Recently, Rousseau-Gueutin et al. [33] showed that the chloroplast acetyl-CoA carboxylase subunit (*accD*) gene present in the plastome of most angiosperms has been functiona.

lly relocated to the nucleus in Campanulaceae, and they experimentally verified the presence of a chloroplastic transit peptide by showing that the product of the nuclear *accD* fused to green fluorescent protein was imported in the chloroplasts. As noted above, Campanulaceae are known to have highly rearranged plastid genomes lacking *accD*, and instead they have an nr-*accD*. Plastid genome information has been thought to mainly depend on studies concerning *T. caeruleum*. More recently, the plastid genomes of *Adenophora remotiflora*, *Campanula takesimana*, and *Hanabusaya asiatica* have been sequenced [41–43]. We have characterized the plastid genome of the Campanulaceae species *Platycodon grandiflorum* cultivar Jangbaek-doraji, as part of a genome project funded by the National Agricultural Genome Center of the Korean Government. As RNA editing information for plastid genes is currently unavailable in Campanulaceae, we also characterized plastid RNA editing in *P. grandiflorum*. To understand plastid genome evolution in Campanulaceae, we have characterized and compared the cp genome and nr-*accD* of *P. grandiflorum* with those of known taxa.

## Results and discussion

### Genome organization and features of *Platycodon grandiflorum*

The general features and plastid genomic structure of *P. grandiflorum* were compared with those of *A. remotiflora*, *C. takesimana, H. asiatica,* and *T. caeruleum* (Campanulaceae) (Tables 1 and 2). The overall GC content of the *P. grandiflorum* genome is low (38.12%), which is similar to that of *A. remotiflora* (38.76%), *C. takesimana* (38.80%), *H. asiatica* (38.76%), and *T. caeruleum* (38.33%). There are 140 genes in the *P. grandiflorum* plastid genome, which is 14 to 19 genes more than identified in *A. remotiflora*, *C. takesimana*, *T. caeruleum,* and *H. asiatica*. The length of the plastid genome in *P. grandiflorum* is 171,818 bp, and the genes account for a coding density of 59.3% of the total cp genome sequence. The latter value is the highest coding density among all reported Campanulaceae species to date. These results indicate that the cp genome of *P. grandiflorum* is more compact than those of the other species considered.

**Table 1:** General features of the plastid genomes in Campanulaceae species

	*Platycodon*	*Hanabusaya*	*Trachelium*	*Campanula*	*Adenophora*
Length	171,818	167,287	162,321	169,551	171,724
GC contents (%)	38.12	38.76	38.33	38.80	38.76
No. of Genes	140	129	126	126	126
No. of Conserved CDS	95	83	80	83	82
No. of tRNA	37	36	36	35	36
No. of rRNA	8	10	10	8	8
No. of Introns	Ι(1) II(24)	Ι(1) II(23)	Ι(1) II(23)	Ι(1) II(23)	Ι(1) II(22)
Coding density	59.35	52.49	46.87	53.60	51.43
CDS + strand	38	44	47	48	40
tRNA + strand	22	18	18	18	20
rRNA + strand	4	5	5	4	4
CDS - strand	57	39	33	35	42
tRNA - strand	15	18	18	17	16
rRNA - strand	4	5	5	4	4
Percent of +/−	45.71	51.94	55.56	55.56	50.79

**Table 2:** Structural comparison of the chloroplast genomes of *Platycodon, Hanabusaya*, *Trachelium, Campanula*, and *Adenophora* of Campanulaceae and other related taxa

Order	Subfamily	Species	Genome size (bp)	LSC size (bp)	IR size (bp)	SSC size (bp)	GenBank Accession number
Asterales	Campanulaceae	*Platycodon grandiflorum*	171,818	79,061	42,433	7840	KX352462
		*Hanabusaya asiatica*	167,287	104,955	26,877	8578	NC_024732
		*Trachelium caeruleum*	162,321	100,110	27,115	7981	NC_010442
		*Campanula akesimana*	169,551	102,320	29,742	7747	NC_026203
		*Adenophora remotiflora*	171,724	105,555	27,437	11,295	NC_02699.1
	Asteraceae	*Helianthus annuus*	151,104	83,530	25,295	17,576	NC_007977
		*Lactuca sativa*	152,765	84,103	25,033	18,596	NC_007578
Apiales	Apiaceae	*Daucus carota*	155,911	84,242	27,050	17,569	NC_008325
		*Anthriscus cerefolium*	154,719	84,774	26,197	17,551	NC_015113
	Araliaceae	*Panax ginseng*	156,318	84,108	26,071	18,070	NC_006290
		*Eleutherococcus senticosus*	156,768	86,755	25,929	18,155	NC_016430

*LSC* large single-copy region, *SSC* small single-copy region, *IR* inverted repeat

In the cp genome of *P. grandiflorum*, there is a biased gene distribution over the two DNA strands, with 38 (40%) conserved genes occupying one strand (+) and 57 genes occupying the other strand (−) (Table 1). The gene contents in one strand were found to be 53, 58, 57, and 48% in the cp genomes of *H. asiatica*, *T. caeruleum*, *C. takesimana* and *A. remotiflora*, respectively. These results indicate that the gene distribution between the two strands of the *P. grandiflorum* cp genome is reversed relative to those of *H. asiatica*, *T. caeruleum*, *C. takesimana* and *A. remotiflora,* which have similar values*.*

**Fig. 1:**
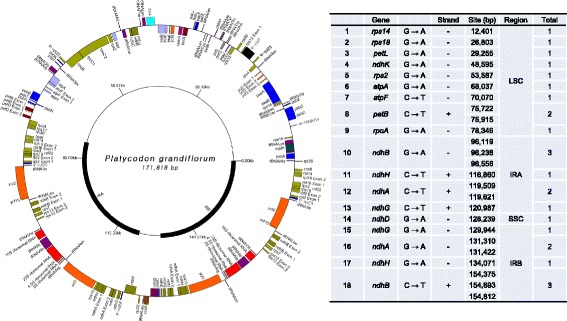
Plastid genome map of *Platycodon grandiflorum* cultivar Jangbaek-doraji showing the RNA editing sites. Genes shown on the outside of the *circle* are encoded on the + strand and transcribed counter-clockwise. Genes on the inside are encoded on the - strand and transcribed clockwise. The relative position of 28 RNA editing sites (19 sites from 14 genes and 7 sites of 4 genes in inverted repeats) is shown in the complete plastid genomic sequence

The plastid genome sequences of *P. grandiflorum* are assembled as circular molecules of 171,818 bp (Fig. 1 and Table 2) containing a 79,061 bp LSC region, a 42,433 bp IR, and a 7840 bp SSC region. The length of the plastid genome in *P. grandiflorum* (171,818 bp) is 2.5–9.5 kb longer than that of *C. takesimana*, *H. asiatica*, and *T. caeruleum*, which are between 162,287 and 169,551 bps in length. Campanulaceae plastid genomes are more than 5 kb longer than the plastid genomes of Asteraceae, Apiaceae, and Araliaceae. The length of the SSC region in Campanulaceae plastid genomes is in the range 7747–8578 bp, which is approximately 10 kb shorter than those of Asteraceae, Apiaceae, and Araliaceae. However, the *P. grandiflorum* plastid genome is distinguished from that of other Campanulaceae species in having a 25 kb longer IR and a 20 kb shorter LSC region.

### Gene contents, RNA editing sites, and cp genome rearrangement in Campanulaecae

The cp genome of *P. grandiflorum* contains 110 genes and 18 introns, comprising 77 protein-coding genes, four RNA genes, 29 tRNA genes, 17 group II introns, and one group I intron (Additional file 1: Table S1). RNA editing of genes was detected in 25 sites of 14 genes, including seven sites in Inverted Repeat B (Fig. 1). Although there was no variation in the RNA editing sites among RNA samples, samples of RNA other than those of leaf RNA lacked some of the gene transcripts depending on the organ of origin (Additional file 2: Table S2). The 14 genes in which RNA editing was detected include ribosomal small subunit genes (*rps2*, *rps14*, and *rps18*), ATP synthase genes (*atpA* and *atpF*), Cytochrome genes (*petB* and *petL*), NADH dehydrogenase genes (*ndhA*, *ndhB*, *ndhD*, *ndhH*, *ndhK*, and *ndhG*), and RNA polymerase gene (*rpoA*). In Asteraceae, a total of 373 editing sites were detected in eight plastid genomes, with the average number of 47 sites per species. Among these, 26 sites of 12 genes were conserved in the eight plastid genomes of Asteraceae [44]. The RNA editing in *rps18*, *atpF*, *petL*, *ndhH*, and *ndhK*, found in *Platycodon*, was not documented in Asteraceae [44].

*P. grandiflorum* lacks five protein-coding genes: *accD*, *clpP*, *infA*, *petE*, and *rpl23*. Of these, *accD*, *clpP*, *infA*, and *rpl23* are also absent in the sequences of the four other Campanulaceae species we examined in this study. *A. remotiflora*, *C. takesimana*, and *T. caeruleum* also lack *petE*, whereas *H. asiatica* has an intact *petE* gene. However, *H. asiatica* lacks *psbE*, which is found in *P. grandiflorum, A. remotiflora, C. takesimana,* and *T. caeruleum*. *C. takesimana, H. asiatica, A. remotiflora,* and *T. caeruleum* lack *ycf15*, which is found in *P. grandiflorum*, whereas *T. caeruleum* lacks *ndhK* and *ycf2*. In addition to the presence/absence of these genes, there is variation in the number of copies of the genes among Campanulaceae species (Table 3). A conspicuous difference found between *P. grandiflorum* and the four other campanule species examined in the present study is variation in the number of copies of *rps12* fragments. Among the 30 conserved *trn* genes, *P. grandiflorum* lacks *trnT_ugu* and *A. remotiflora, C. takesimana,* and *H. asiatica* lack *trnT_ggu*, whereas *T. caeruleum* has all 30 *trn* genes (Additional file 3: Table S3). As a minimal set of plastid *trnA* genes [45, 46] for trytophan, *P. grandiflorum* uses only *trnT_ggu*, whereas *A. remotiflora, C. takesimana,* and *H. asiatica* use only *trnT_ugu*. In contrast, *T. caeruleum* uses both *trnT_ggu* and *trnT_ugu* for trytophan in the plastid genome.

**Table 3:** Plastid protein-coding gene distribution among Campanulaceae species

	*Platycodon*	*Hanabusaya*	*Trachelium*	*Campanula*	*Adenophora*		*Platycodon*	*Hanabusaya*	*Trachelium*	*Campanula*	*Adenophora*
*accD*	−	−	ΨΨ	−	−	*psbH*	+	+	+	+	+
*atpA*	+	+	+	+	+	*psbI*	+	+	+	+	+
*atpB*	+	+	+	+	+	*psbJ*	+	+ +Ψ	+ + +	+ +Ψ	+ +Ψ
*atpE*	+	+	+	+	+	*psbK*	+	+	+	+	+
*atpF*	+	+	+	+	+	*psbL*	+	+	+	+	+
*atpH*	+	+	+	+	+	*psbM*	+	+	+	+	+
*atpI*	+	+	+	+	+	*psbN*	+	+	+	+	+
*ccsA*	+	+	+	+	+	*psbT*	+	+	+	+	+
*cemA*	+	+	+	+	+	*psbZ*	+	+	+	+	+
*clpP*	Ψ	ΨΨ	ΨΨ	ΨΨ	ΨΨ	*rbcL*	+	+	+	+	+
*infA*	−	−	Ψ	Ψ	Ψ	*rpl14*	+ +	+	+	+	+
*matK*	+	+	+	+	+	*rpl16*	+ +	+	+	+	+
*ndhA*	+ +	+ +	+ +	+ +	+ +	*rpl2*	+ +	+	+	+	+
*ndhB*	+ +	+ ^**1**^	+	+	Ψ	*rpl20*	+	+	+	+	+
*ndhC*	+	+	+	+	+	*rpl22*	+ +	+	+	+	+
*ndhD*	+	+	+	+	+	*rpl23*	ΨΨΨΨ	Ψ	Ψ	Ψ	Ψ
*ndhE*	+Ψ	+	+Ψ	+Ψ	+Ψ	*rpl32*	+	+	+	+	+
*ndhF*	+	+	+	+	+	*rpl33*	+	+	+	+	+
*ndhG*	+ +	+ +	+ +	+ +	+ +	*rpl36*	+ +	+	+	+	+
*ndhH*	+ +	+ +	+ +	+ +	+ +	*rpoA*	+	+	+	+	+
*ndhI*	+ +	+ +	+	+ +	+ +	*rpoB*	+	+	+	+	+
*ndhJ*	+	+	+	+	+	*rpoC1*	+	+	+	+	+
*ndhK*	+	+	Ψ	+	+	*rpoC2*	+	+	+	+	+
*petA*	+	+	+	+	+	*rps11*	+	+	+	+	+
*petB*	+	+	+	+	+	*5′ rps12*	+	+ +	+ +	+ +	+ +
*petD*	+	+	+	+	+	*3′ rps12*	+ +	+	+	+	+
*petE*	−	+	−	−	−	*rps14*	+	+	+	+	+
*petG*	+	+	+	+	+	*rps15*	+ +	+ +	+ +	+ +	+ +
*petL*	+	+	+	+	+	*rps16*	+ ^**2**^	+	+	+	+
*petN*	+	+	+	+	+	*rps18*	+	+	+	+	+
*psaA*	+	+	+	+	+	*rps19*	+ +	+	+	+	+
*psaB*	+	+	+	+	+	*rps2*	+	+	+	+	+
*psaC*	+	+	+	+	+	*rps3*	+ +	+	+	+	+
*psaI*	+	+	+	+	+	*rps4*	+	+	+	+	+
*psaJ*	+	+	+	+	+	*rps7*	+ +	+	+	+	+
*psbA*	+	+	+	+	+	*rps8*	+ +	+	+	+	+
*psbB*	+	+ΨΨ	+ΨΨ	+ΨΨ	+ΨΨ	*ycf1*	+ +	+ +	+	+ +	+ +
*psbC*	+	+	+	+	+	*ycf2*	+ +	+	Ψ	+	+
*psbD*	+	+	+	+	+	*ycf3*	+	+Ψ	+ΨΨ	+ΨΨ	+ΨΨ
*psbE*	+	−	+	+	+	*ycf4*	+	+	+	+	+
*psbF*	+	+	+	+	+	*ycf15*	+ +	−	Ψ	Ψ	−

‘+’ indicates presence of the gene, ‘Ψ’ indicates pseudo-copy of the gene, and ‘-’ indicates complete absence of the gene. ‘ΨΨ’ indicates two pseudo-copies of the gene

1, 5′ end variation; 2, GTG starting codon

Angiosperm plastids generally contain one group I intron and 20 group II introns. The *P. grandiflorum* cp genome was found to contain 17 different introns, including 16 group II introns and one group I intron with a cyanobacterial origin [47] located within the *trnL_uaa* gene (Additional file 4: Table S4). Three protein-coding genes, *clpP*, *rps12*, and *ycf3*, contain two group II introns (*rps12.i1*, *rps12.i2*, *ycf3.i1* and *ycf3.i2*), and 14 genes contain a single group II intron: *rpl2.i, rpl16.i, atpF.i, petB.i, petD.i, ndhA.i, ndhB.i, trnA_ugc.i, trnG_ucc.i, trnI_gau.i, trnK_uuu.i*, and *trnV_uac.i*. Of the 20 group II introns, the intron in *rps12*, between exons 1 and 2, is *trans*-splicing, whereas the other 19 group II introns are *cis*-splicing.

Thirty genes, six introns, and parts of two genes and one intron are found within the IR, which has two copies. These 19 genes include seven protein-coding genes (*ndhA*, *ndhB, ndhG, ndhH, ndhI, rpl2, rpl14, rpl16, rpl22, rpl36, rps3, rps7, rps8, rps12.e2, rps12.e3, rps15, rps19, ycf1, ycf2,* and *ycf15*), all four rRNA genes (16S, 23S, 4.5S, and 5S), and seven tRNA genes (*trnA_ugc, trnI_cau, trnI_gau, trnL_caa, trnN_guu, trnR_acg,* and *trnV_gau*). The six introns are *ndhB.i, rpl2.i, trnA_ugc.i, trnI_gau.i*, *rps12.i1t,* and *rps12.i2*. The IR also contains the 5′ end of *ndhE* at the border with the SSC region, resulting in one intact *ndhE* and a 192 bp *ψ-ndhE* in the cp genome. In addition, the IR contains parts of the *rps12* gene. This *rps12* gene consists of three exons, *rps12.e1*, *rps12.e2*, and *rps12.e3* [48]. *rps12.e1* is located in the LSC region, whereas *rps12.e2* and *rps12.e3* are located in the IR. Thus, the genome contains a single copy of *rps12.e1* but has two copies of *rps12.e2* and *rps12.e3*. A *cis*-splicing group II intron, *rps12.i2*, intervenes between *rps12.e2* and *rps12.e3*, but a *trans*-splicing intron, *rps12.i1t*, occurs between *rps12.e1* and *rps12.e2*. The *rps12.i1t* is split into two sections, *rps12.i1t1* and *rps12.i1t2*. This is because the *rps12* gene is transcribed in two separate operons, namely, the *clpP* operon (*ψ-clpP* - *rps12.e1* - *rps12.i1t1* - *rpl20*) and the 3′ *rps12* operon (*rps12.i1t2* - *rps12.e2* - *rps12.i2* - *rps12.e3* - *rps7-ndhB*). *P. grandiflorum* contains the 3′ *rps12* operon (*rps12.i1t2* - *rps12.e2* - *rps12.i2* - *rps12.e3* - *rps7* - *ndhB*) within the IR.

**Fig. 2:**
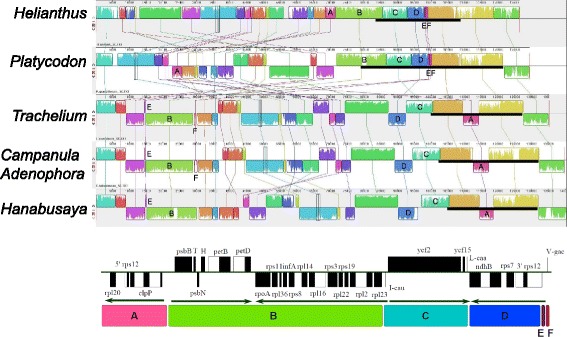
Comparison of the chloroplast genomes of *Helianthus* of Asteraceae and *Platycodon, Trachelium, Adenophora, Campanula*, and *Hanabusaya* of Campanulaceae. In this analysis, Inverted Repeat B was removed for convenience. A conserved region was found at the junction between the large single-copy (LSC) region and the IR of the angiosperm plastid genome, but it was rearranged in the Campanulaceae plastids. The *thick black bar* indicates the IR region of each species. Regions are marked in six parts, **a-f**. **a**: *clpP* operon (− strand); **b**: *psbB* operon (+ strand) - *rpl23* operon (+ strand); **c**: *ycf2* - *ycf15* - *trnL_caa* (+ strand); D: 3′ *rps12* operon (− strand); **e**: Intergenic spacer between 3′ *rps12* and *trnV_gac* genes; **f**: *trnV_gac* gene (+ strand)

The complete cp genomes of *P. grandiflorum, C. takesimana, H. asiatica,* and *T. caeruleum* of Campanulaceae and *Helianthus annuus* of Asteraceae were compared using the MAUVE alignment tool. Inverted Repeat B of the five chloroplasts was deleted prior to the MAUVE alignment, such that the genome-level alignments could be maximally shown (Fig. 2). In Fig. 2, the thick black bar indicates the IR region of each species. *Helianthus* has a typical asterid chloroplast genomic structure. A comparison of the cp-DNAs of *Helianthus* and *Platycodon* shows the expansion of the IR toward both LSC and SSC in *Platycodon* (Fig. 2 and Table 2), whereas comparison among the campanule cp-genomes shows disruption of the IR and LSC by severe rearrangement.

The region between the LSC region and the IR of angiosperm plastid genomes is generally conserved: 5′ - (*clpP* operon) - *psbB* operon [49] - (*rpl23* operon) - *ycf2* - *ycf15* - *trnV_gac* - (3′ *rps12* operon) - 3′ (Fig. 2). In contrast to *Helianthus,* the *clpP* operon (A) of the LSC region has relocated to the middle of the LSC region in *P. grandiflorum*. However, the *clpP* operon (A) has relocated within the IR in the cp-DNA of *A. remotiflora*, *C. takesimana, H. asiatica,* and *T. caeruleum*. In these four taxa, fragments B, D (3′ *rps12* operon), E, and F have relocated in the middle of the LSC region.

**Fig. 3:**
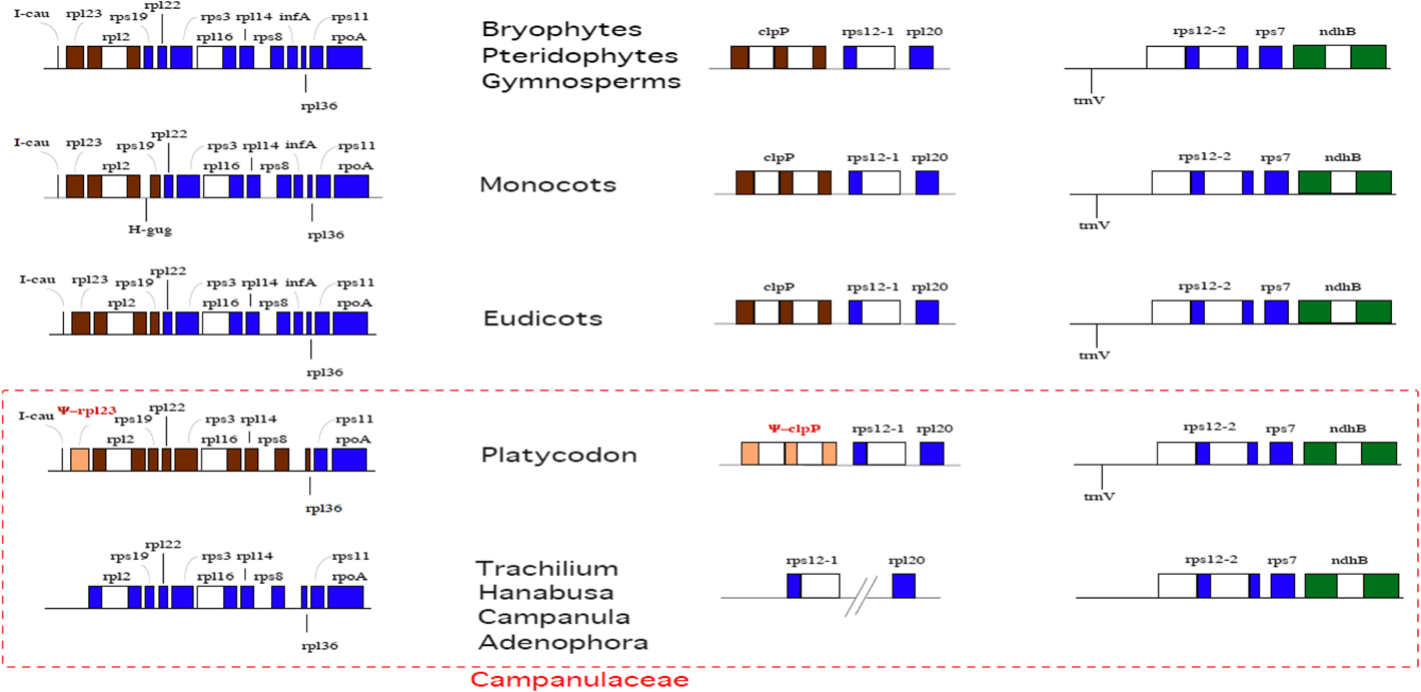
Comparison of the *rpl23*, *clpP*, and 3′ *rps12* gene clusters in land plant plastids. Genes shown on the upper part are encoded on the + strand and genes shown on the lower part are encoded on the - strand. *Closed boxes* indicate exons and *open boxes* indicate introns. The left column shows the *rpl23* operon. The *dark brown* color indicates inverted repeats (IR) and *blue* color indicates the large single-copy region (LSC). The middle column shows the *clpP* operon. *Platycodon* has an *clpP* operon structure with a trace of exons and introns. Other known Campanulaceae species have lost the *clpP* operon structure by genomic rearrangement. The right column shows the 3′ *rps12* operon. *trnV_gac* precedes the 3′ *rps12* operon in *Platycodon* and most land plants, but other known Campanulaceae species have lost this arrangement

The *rpl23* gene cluster containing 13 genes (*trnI_cau* - *rpl23* - rpl2* - *rps19* - *rpl22* - *rps3* - rpl16* - *rpl14* - *rps8* - *infA* - *rpl36* - *rps11* - *rpoA*) (Fig. 3) is conserved from the charophyte *Spirogyra* [48, 50], bryophytes [51], ferns [52], and gymnosperms [53] to eudicots. *P. grandiflorum* has lost *rpl23* and *infA*. In *A. remotiflora*, *C. takesimana, H. asiatica,* and *T. caeruleum*, *trnI_cau* has been relocated in the LSC region, separate from the *rpl23* gene cluster. The *clpP* operon (clpP** - *rps12.e1* - *rps12.i1t1* - *rpl20*) is conserved from bryophytes [51], ferns [52], and gymnosperms [53] to eudicots. *P. grandiflorum* carries *ψ-clpP* - *rps12.e1* - *rps12.i1t1* - *rpl20. A. remotiflora*, *C. takesimana, H. asiatica,* and *T. caeruleum* harbor a member of the *clpP* operon divided by genomic rearrangement into two separated fragments, *rps12.e1* - *rps12.i1t1* and *rpl20*. The structure of the (*trnV_gac*) - 3′ *rps12* operon in the IR is conserved from *Spirogyra* [48], bryophytes [51], ferns [52], gymnosperms [53], and eudicots to *P. grandiflorum. A. remotiflora*, *C. takesimana, H. asiatica,* and *T. caeruleum* exhibit the members of this structure divided into two separated fragments, *trnV_gac* and the 3′ *rps12* operon in the LSC region. The results indicate that the cp-DNA of *A. remotiflora*, *C. takesimana, H. asiatica,* and *T. caeruleum* is somewhat derived compared with that of *P. grandiflorum.*

The duplicative nature of the IR reduces the substitution rate within this region. As the most illustrative single example of the effect of IR duplication on substitution rates, Zhu et al. [13] demonstrated that, consistent with other comparisons, the SC-to-IR genes in *Hanabusaya* and *Trachelium* show IR-like substitution rates, whereas their IR-to-SC genes show SC-like substitution rates. However, in the case of *Platycodon*, the SC-to-IR genes have been generated by a border shift, rather than genomic rearrangement, as shown in other campanules.

### Nr-*accD* of *P. grandiflorum*

**Fig. 4:**
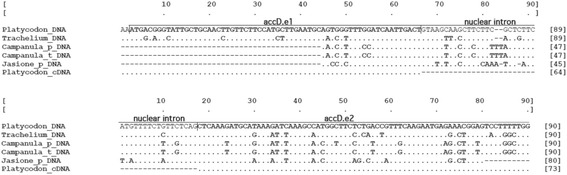
Border sequences between the nr-*accD* exons and introns in Campanulaceae species. The nr-*accD* of Campanulaceae contains a nuclear intron. The first row shows the aligned border of the *accD.e1* and *accD* introns and the second row shows the aligned border of *accD* and *accD.e2* introns. The dot indicates the same sequence of the first line sequence and ‘-’ indicates absence of the sequence or gap

A 1282 bp segment of nr-*accD* mRNA containing a 996 bp exon was recovered from RNA seq reads. The sequence was verified via RT-PCR, followed by sequencing. Genomic DNA sequences for *nr-accD* with lengths of 225 bp and 1464 bp were recovered from DNA-seq reads, referenced according to the 1282 bp mRNA sequence. We recovered a 4.1 kb sequence of the genomic *nr-acc*D gene fragment. The cDNA and DNA sequences of *P. grandiflorum* nr-*accD* were compared with the previously reported nr-*accD* gene and intron sequences of *T. caeruleum* (JQ693029), *Jasione perennis* (JQ693031), *Campanula thyrsoides* (JQ693032), and *Campanula punctate* (JQ693033) [33]*.* The intron of *nr-accD* in *P. grandiflorum* has the same insertion site as observed in these other species (Fig. 4). About 3.2 kb intron of *P. grandiflorum* nr-*accD* (Additional file 5: Figure S1) appears to be the largest among the taxa examined to date: *Jasione perennis* (2250 bp), *T. caeruleum* (1358 bp), *Campanula thyrsoides* (2177 bp), and *Campanula punctate* (2431 bp). These results indicate that the campanule nr-*accD* and its intron share a common ancestor.

### Phylogeny of plastids and *accD* genes among Campanulaceae species

**Fig. 5:**
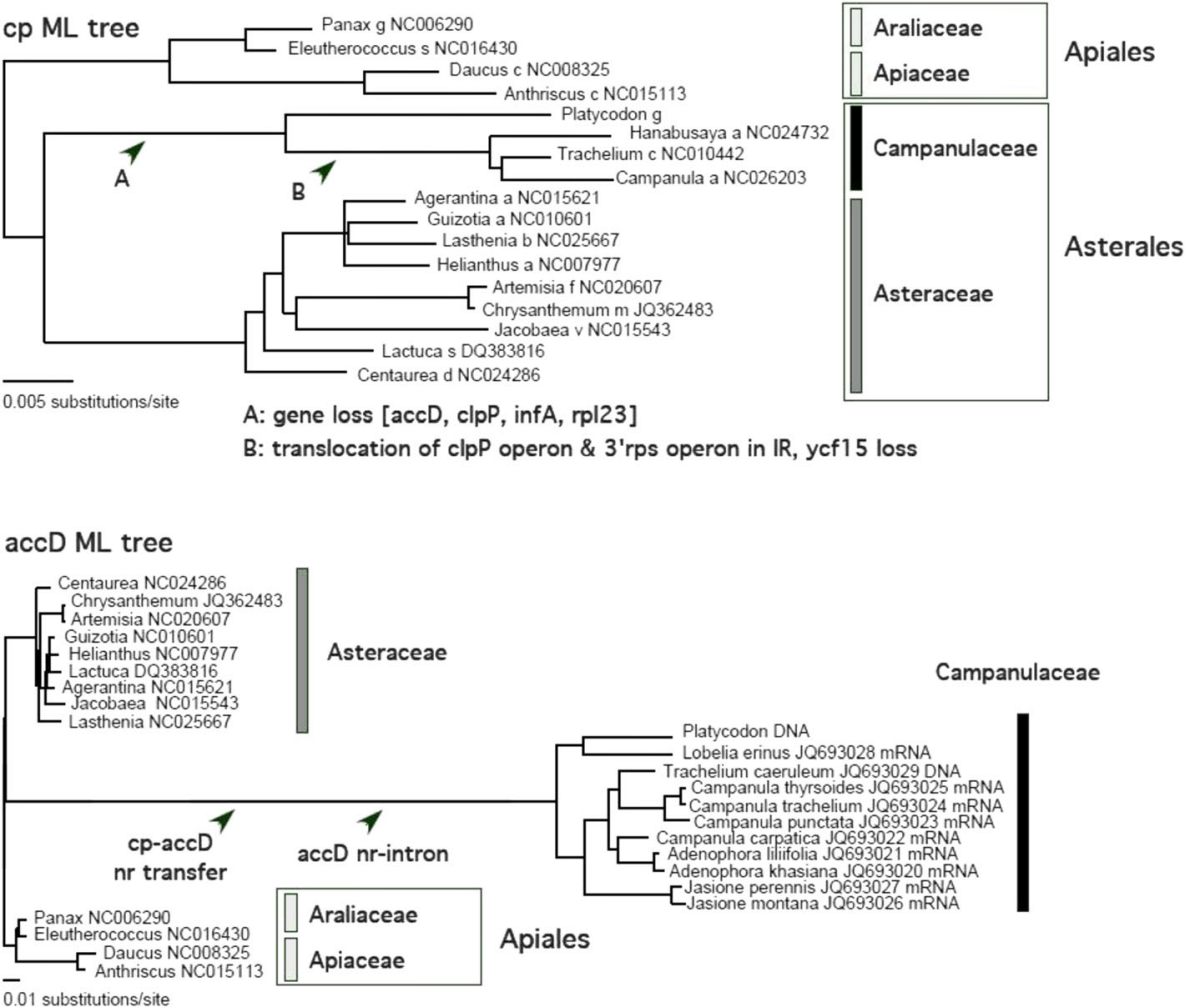
Single maximum-likelihood (ML) trees of plastid genes and the *accD* gene. A: Single ML (HYK85 + G + I model) tree based on the DNA sequences of seven cp genes (six photosystem genes and *rbcL*). A: Single ML (HYK85 + G + I model) tree based on the DNA and RNA sequence of nr-*accD* gene and cp-*accD* gene

Phylogenetic relationships among the plastids of four Campanulaceae species were investigated using the aligned 10,950 bp DNA sequence of seven large photosystem genes - *psaA*, *psaB*, *psbA*, *psbB*, *psbC*, and *psbD*, and *rbcL*. The seven genes representing the plastid genome in the phylogeny [43] are without RNA editing, which might affect phylogenetic topology. Using maximum parsimonious (MP), and neighbor-joining (NJ), and maximum likelihood (ML) methodologies, phylogenetic analysis outgrouped by 13 taxa produced single plastid trees with similar topologies (Additional file 6: Figure S2). The plastid phylogeny showed that *P. grandiflorum* is the most basal of the clade of Campanulaceae species (Fig. 5).

Phylogenetic relationships among 11 Campanulaceae nr-*accD* genes were investigated using the aligned 616 bp DNA and RNA sequences. Using MP, NJ, and ML analyses, phylogenetic analysis outgrouped by cp-*accD* sequences from 13 taxa produced single plastid trees with similar topologies (Additional file 7: Figure S3). The *accD* phylogeny showed that *P. grandiflorum* and *Lobelia erinus* formed the basal-most clade from the lineage of nine taxa (Fig. 5). The results of both phylogenies indicate that *P. grandiflorum* is a basal lineage of Campanulaceae.

### The evolution of *accD* and cpDNA in Campanulaceae

Phylogenetic study of nr-*accD* and cp-*accD* (Fig. 5) indicates that the nr-*accD* of Campanulaceae is of single origin. The cp-*accD* was transferred to the nucleus in the early campanules and later a nuclear intron was introduced. The phylogeny of cpDNA indicates that IR expansion and the loss of four cp-genes (*accD*, *clpP*, *infA*, and *rpl23*), represented by *Platycodon* cp-DNA, had occurred in the early campanule plastid genome, followed by the translocation of the *clpP* and 3′ *rps12* operons between the LSC and IR regions. The evolution of an IR with 30 genes would have slowed down the evolutionary speed in the early campanule. In this regard, further characterization of basal campanules, including Lobeliaceae, would enable us to gain a better understanding of cp-genome evolution.

Among embryophytes, in 80% of the cases where *ycf1* was lost from the plastid genome, there was a concomitant loss of *accD* [54, 55]. In *Odontella purpurea*, *Erodium* of Generaniaceae and *Vaccinium macrocarpon* of Ericaceae, *accD* is still present in the chloroplast genome, although it does not encode a YCF1 homolog [55]. All five campanules investigated in the present study, including *Platycodon*, lack cp-*accD* and the complete *ycf1* gene is within the IR, in which synonymous substitution rates are, on average, 3.7 times slower than those in SC genes [13]. The substitution rate of *accD* and *ycf1*, both of which are located in the SC region of most angiosperms, is high [55, 56]. The results may indicate that YCF1 is involved in the assembly of the ACCase holoenzyme [55].

## Conclusions

In this study, we characterized the 171,818 bp cp genome of *P. grandiflorum*, the largest among known Campanulaceae species. This genome contains 110 genes and 18 introns, among which there are 77 protein-coding genes, four RNA genes, 29 tRNA genes, 17 group II introns, and one group I intron. RNA editing of genes was detected in 18 sites of 14 genes. *P. grandiflorum* cp-DNA lacks five protein-coding genes, namely, *accD*, *clpP*, *infA*, *petE*, and *rpl23*. Of these, we characterized nr-*accD*. *Platycodon* nr-*accD* contains about 3.2 kb intron between nr-*accD.e1* (64 bp) and nr-*accD.e2* (932 bp) at the same insertion position as in other Campanulaceae. Unlike the highly rearranged cp-DNAs of *A. remotiflora*, *C. takesimana, H. asiatica,* and *T. caeruleum*, *P. grandiflorum* cp-DNA contains the 5′ - *psbB* operon - (*rpl23* operon) - *ycf2* - *ycf15* - *trnV_gac* - (3′ *rps12* operon) - 3′, which is conserved in most land plant plastid genomes. Phylogenetic studies of cp genes and *accD* genes support the hypothesis that *P. grandiflorum* belongs to the basal lineage of Campanulaceae.

Our phylogenetic studies also support the notion that severe genomic rearrangements occurred in the chloroplast genome after *accD* nuclear transfer and the loss of four genes in early Campanulaceae as a lineage of asterids. *accD, clpP, infA*, and *rpl23* are also absent in all three known Campanulaceae species. The loss of these four genes in the cp genome appears to be a shared derived characteristic in Campanulaceae. Further survey of the cp genomes of Campanulaceae and their close relatives will provide a better understanding of the nuclear transfer of the members of cp genomes.

## Methods

### Plant materials and nucleotide extraction

*P. grandiflorum* cultivar Jangbaek-doraji was grown for 1 year in a bellflower field in the Department of Herbal Crop Research, RDA, Eumseong, Korea. Collected samples were divided into leaves, stems, roots, petals, sepals, pistils, stamens, and seeds. To extract total RNA, each sample was frozen in liquid nitrogen and ground using a mortar and pestle. Total DNA was extracted using a DNeasy Plant Mini Kit (Qiagen, USA), and total RNA for cDNA library construction was extracted using an RNeasy Plant Mini Kit (Qiagen, USA) according to the manufacturer’s instructions.

### Sequencing, assembly, and annotation of the *Platycodon* plastid genome

The plastid genome of *P. grandiflorum* cultivar Jangbaek-doraji was sequenced as part of the Jangbaek-doraji genome project (funded by the National Agricultural Genome Center). Three Illumina paired-end (PE) genomic libraries of 270, 500, and 700 bp were constructed and sequenced using an Illumina HiSeq 2000 platform. The plastid sequence was obtained using CLC Genomics Workbench version 8.0. The circular structures of each replicon were confirmed by polymerase chain reaction (PCR) amplification at their ends and by joining of Sanger sequence reads derived from the amplicons. The assemblies were further verified by examining paired-end distance and depth after re-mapping reads on the contig sequences. BLAST searches of a large contig were verified to be plastid genomes. For gene annotation of organelle genomes, protein-coding and ribosomal RNA genes were annotated using DOGMA (http://dogma.ccbb.utexas.edu/) [57]. The boundaries of each annotated gene were manually determined by comparison with orthologous genes from other known cp genomes. Genes encoding tRNAs were initially predicted using tRNAscan (http://lowelab.ucsc.edu/tRNAscan-SE) [58] and ARAGORN version 1.2 (http://130.235.46.10/ARAGORN/) [59], and were then manually verified by predicting the tRNA secondary structure. Circular genome maps were drawn using GenomeVx [60], followed by manual modification. The sequencing data and gene annotations were submitted to GenBank with accession number KX352464.

### RNA sequencing and RNA editing site tracing from the plastid genome

The quality of the resulting total RNA was measured using an Agilent 2100 Bioanalyzer (Agilent Technologies, Santa Clara, CA). All extractions delivered an RNA integrity number value (RIN) of >7.0 and a 28S:18S ratio ≥ 1.5.

Poly-A-containing mRNA molecules were purified from 2 μg of total RNA of each sample using poly-T oligo-attached magnetic beads. The mRNA was fragmented into an insert size of approximately 200 bp. The first-strand cDNA of the mRNA fragments was synthesized using reverse transcriptase and random hexamer primers. The second-strand cDNA was then synthesized using DNA Polymerase I and RNaseH to generate double-stranded cDNA. These cDNA fragments then went through an end repair process, the addition of a single “A” base, and ligation of adapters. The products were then purified and enriched by PCR to amplify the amount of DNA in the library. The libraries were quantified using a KAPA library quantification kit (KAPA Biosystems, South Africa) in an Agilent 2100 Bioanalyzer (Agilent Technologies, Waldbronn, Germany). After qPCR validation, libraries were subjected to paired-end sequencing with a 100 bp read length using the Illumina HiSeq 2500 platform (Illumina). After the completion of a sequencing run, raw image files were processed using Illumina Real-Time Analysis (RTA) for image analysis and base calling. Raw data were saved as FASTQ files. Clean reads were obtained by removing adaptor sequences, reads in which the percentage of unknown bases (N) was greater than 10%, and low-quality reads (more than 20% < Q20 bases). The high-quality reads were directly mapped to the plastid genome to trace RNA editing sites.

### mRNA sequencing, cDNA synthesis, RT-PCR, and DNA-PCR for nr-*accD*

The RNA-seq analysis results were analyzed using CLC Genomics Workbench 8.0 (CLC bio, Denmark). The adapter sequences contained in the data were removed using the trim sequence program and remaining sequences were assembled into contigs by de novo assembly. The plastid *accD* of *Helianthus* was used to find the nr*-acc*D of *P. grandiflorum* using an Xblast search. A 1282 bp RNA seq containing a 1020 bp nr*-accD* was found. cDNA was synthesized from 1 μg of total RNA, which was extracted from a *P. grandiflorum* leaf using Plant TRI reagent (Invitrogen, USA). cDNA was synthesized using an iScriptTM cDNA Synthesis Kit (Bio-Rad, USA) and a 2720 Thermal Cycler (Applied Biosystems, USA) according to the manufacturers’ instructions. PCR amplification of the *accD* gene was carried out using the HS PrimeSTAR Component Mixture (Takara, Japan). The PCR reaction consisted of a total of 50 μL (10 μL of 5× HS buffer, 1 μL of forward and reverse primers (10 μM, Forward; 5′-GAGAGAAATGACGGGTATTGC-3′, Reverse; 5′-CTCCCACTCAAAATGTTTTAC-3′), 5.0 μL of dNTP, 1.0 μL of template, 2.5 units of PrimeSTAR polymerase, and made to volume with distilled water). The amplification program was as follows: preheating at 98 °C for 1 min, followed by 28 cycles of denaturation at 98 °C for 10 s, annealing at 58 °C for 30 s, and extension at 72 °C for 1 min and 30 s, and a final extension at 72 °C for 5 min. The 1240 bp PCR product was purified using a Biomedical Gel and PCR Purification Kit (Biomedic, Korea) and sequenced using a 3730 DNA Analyzer (Applied Biosystems, USA).

Genomic DNA sequences of *nr-accD* with lengths of 225 bp and 1464 bp were recovered by mapping DNA reads to the 1282 bp RNA sequence using CLC Genomics Workbench version 8.0. A 4.2 kb genomic *accD* gene fragment was recovered using LA Taq (Takara, Japan), and two long primer sets: *accD*_PL_LPRF (5′-GGTATTGCTGCAACTTGTTCTTCCATGC-3′) and *accD*_PL_LPR (5′-TCTCGAACAAATACTCGGCCTGTTGTACGC-3′); *accD*_PL_LPRF1 (5′- ACTTGTTCTTCCATGCTTGAATGCAGTGG-3′) and *accD*_PL_LPR1 (5′- CCAGCATAGCGAAACTAGCTGTCACCCCTC-3′). The RNA sequence for nr-*accD*, two DNA sequences containing nr-*accD.e1*, nr-*accD.e2*, and partial sequences of the nr-*accD* intron were submitted to GenBank with the accession numbers KX352462 and KX352463. The cDNA and DNA sequences of *P. grandiflorum* nr-*accD* were compared with the previously reported nr-*accD* gene and intron DNA sequences of *T. caeruleum* (JQ693029), *Jasione perennis* (JQ693031), *Campanula thyrsoides* (JQ693032), and *Campanula punctate* (JQ693033) [27]*.*

### Comparative analysis of cp genomes

The complete cp genomes of *P. grandiflorum, C. takesimana, H. asiatica,* and *T. caeruleum* of Campanulaceae and *Helianthus annuus* of Asteraceae were compared using the MAUVE alignment tool [61] to identify rearrangement-free locally collinear blocks (LCBs) among genomes, yielding 25 LCBs with a minimum weight of 170. Inverted repeat A of the five chloroplasts was deleted prior to the MAUVE alignment so that the genome-level alignments could be maximally shown.

### Phylogenetic analysis

The phylogenetic relationships of Campanulaceae in Asterales were investigated using the chloroplast genomic information. Reported Campanulaceae plastid genomic information for *H. asiatica* (NC024732), *C. takesimana* (NC026203), and *T. caeruleum* (NC010442) was included as an ingroup. To avoid bias by taxon sampling, nine Asteraceae, two Apiaceae, and two Araliaceae were used as an outgroup. These included *Agerantina* (NC015621), *Guizotia* (NC010601), *Helianthus* (NC007977), *Lasthenia* (NC025667), *Artemisia* (NC020607), *Chrysanthemum* (JQ362483), *Jacobaea* (NC015543), *Lactuca* (DQ383816), and *Centaurea* (NC024286) of Asteraceae; *Daucus* (NC008325) and *Anthriscus* (NC015113) of Apiaceae; and *Panax* (NC006290) and *Eleutherococcus* (NC016430) of Araliaceae. DNA sequences of seven cp protein genes, *psaA*, *psaB*, *psbA*, *psbB*, *psbC*, *psbD*, and *rbcL*, were used to construct cp phylogenetic trees by MP, NJ, and ML analyses using Paup ver. 6.0 [62]. In addition, the phylogenetic relationships of *accD* were investigated. cp-*accD* sequences from 13 taxa were used as an outgroup, and all reported Campanulaceae nr-*accD* RNA and DNA sequences were included as an ingroup. These included the sequences of *Lobelia erinus* (JQ693028), *T. caeruleum* (JQ693029), *Campanula thyrsoides* (JQ693025), *Campanula trachelium* (JQ693024), *Campanula punctata* (JQ693023), *Campanula carpatica* (JQ693022), *Adenophora liliifolia* (JQ693021), *Adenophora khasiana* (JQ693020), *Jasione perennis* (JQ693027), and *Jasione montana* (JQ693026) [33].

## Additional files

Additional file 1: Table S1. Gene contents of the *Platycodon grandiflorum* plastid. (DOCX 25 kb)

Additional file 2: Table S2. Cytidine (C) to uridine (U) editing sites in the chloroplast genome of *Platycodon* validated by RNA-Seq data from leaf, root, stem, seed, petal, pistil, sepal, and stamen. (DOCX 21 kb)

Additional file 3: Table S3. Plastid tRNA and rRNA gene distribution among Campanulaceae species. ‘+’ indicates presence of the gene, and ‘-’ indicates complete absence of the gene. ‘ΨΨ’ indicates two pseudo-copies of the gene. The number of ‘+’ indicates the copy number of the gene. (DOCX 16 kb)

Additional file 4: Table S4. Plastid intron distribution in Campanulaceae species and other related taxa. ‘+’ indicates presence of the gene, ‘Ψ’ marks pseudo-copy of the gene, and ‘-’ indicates complete absence of the gene. ‘ΨΨ’ indicates two pseudo-copies of the gene. (DOCX 18 kb)

Additional file 5: Figure S1. cDNA and DNA PCR confirmation of nr-*accD* in *Platycodon grandiflorum*. A: The size of the nr-*accD* cDNA sequence, primer sites, and cDNA PCR products in *P. grandiflorum* cultivars. B: The size of the nr-*accD* DNA sequence, primer sites, and genomic DNA PCR products in *P. grandiflorum* cultivars. PL03 [*accD*_PL_LPRF ~ *accD*_PL_LPR] and PL05 [*accD*_PL_LPRF1 ~ *accD*_PL_LPR1]. (PPTX 757 kb)

Additional file 6: Figure S2. Phylogenetic trees generated from the DNA sequences of seven cp-genes using three algorithms. (A) single maximum parsimonious (MP) tree, (B) single neighbor-joining (NJ) tree, and (C) single maximum likelihood (ML) tree (HYK85 + G + I model). (PPTX 749 kb)

Additional file 7: Figure S3. Phylogenetic trees generated from the DNA sequences of the *accD* gene using three algorithms. (A) single maximum parsimonious (MP) tree, (B) single neighbor-joining (NJ) tree, and (C) single maximum likelihood (ML) tree (HYK85 + G + I model). (PPTX 857 kb)

## Abbreviations

3′ *rps12* operon: *rps12.i1t2* - *rps12.e2* - *rps12.i2* - *rps12.e3* - *rps7*
*clpP***: *clpP* having two introns
cp: Chloroplast
IRs: Inverted repeats
LSC: Large single-copy region
nr: Nuclear
*rpl2**: *rpl2* having an intron
*rpl2.i*: An intron in *rpl2*
*rps12.e1*: Exon 1 of *rps12*
*rps12.i1t*: Trans-spliced first intron in *rps12*
*rps12.i1t1*: 5′ end sequence of trans-spliced first intron in *rps12*
*rps12.i1t2*: 3′ end sequence of trans-spliced first intron in *rps12*
*rps12.i2*: The second intron of *rps12*
SSC: Small single-copy region
