# Supplementary material for: accD nuclear transfer of Platycodon grandiflorum and the plastid of early Campanulaceae
Source: BMC Genomics. 2017 Aug 11;18:607. doi: 10.1186/s12864-017-4014-x (PMC5553655; doi:10.1186/s12864-017-4014-x)
Supplement: Supplementary file 1 — Gene contents of the Platycodon grandiflorum plastid. (DOCX 25 kb) [file 12864_2017_4014_MOESM1_ESM.docx]

Table S1

|  | **Gene** | | | | | | | | |
| --- | --- | --- | --- | --- | --- | --- | --- | --- | --- |
| Photosystem Ⅰ | *psaA* | *psaB* | *psaC* | *psaI* | *psaJ* | *ycf3*** |  |  |  |
| Photosystem II | *psbA* | *psbB* | *psbC* | *psbD* | *psbE* | *psbF* | *psbH* | *psbI* | *psbJ* |
|  | *psbK* | *psbL* | *psbM* | *psbN* | *psbT* | *psbZ* |  |  |  |
| Cytochrome b6/f complex | *petA* | *petB** | *petD** | *petG* | *petL* | *petN* |  |  |  |
| ATP synthase | *atpA* | *atpB* | *atpE* | *atpF** | *atpH* | *atpI* |  |  |  |
| Rubisco large subunit | *rbcL* |  |  |  |  |  |  |  |  |
| C-type cytochrome synthesis | *ccsA* |  |  |  |  |  |  |  |  |
| NADH dehydrogenase | *ndhA** x 2 | *ndhB** x 2 | *ndhC* | *ndhD* | *ndhE* | *ndhF* | *ndhG* x 2 | *ndhH* x 2 | *ndhI* x 2 |
|  | *ndhJ* | *ndhK* |  |  |  |  |  |  |  |
| RNA polymerase | *rpoA* | *rpoB* | *rpoC1* | *rpoC2* |  |  |  |  |  |
| Maturase K | *matK*^β^ |  |  |  |  |  |  |  |  |
| Large subunit ribosomal proteins | *rpl2** x 2 | *rpl14* x 2 | *rpl16** x 2 | *rpl20* | *rpl22* x 2 | *rpl32* | *rpl33* | *rpl36* x 2 |  |
| Small subunit ribosomal proteins | *rps2* | *rps3* x 2 | *rps4* | *rps7* x 2 | *rps8* x 2 | *rps11* | *rps12***^α^ x 2 | *rps14* | *rps15* x 2 |
|  | *rps16* | *rps18* | *rps19* x 2 |  |  |  |  |  |  |
| Envelope membrane protein | *cemA* |  |  |  |  |  |  |  |  |
| Conserved orf | *ycf1*^γ^ x 2 | *ycf2* x 2 | *ycf15* x 2 |  |  |  |  |  |  |
| Ribosomal RNA | *rrn16S* x 2 | *rrn23S* x 2 | *rrn4.5S* x 2 | *rrn5S* x 2 |  |  |  |  |  |
| tRNAs | *trnA_ugc** x 2 | *trnC_gca* | *trnD_guc* | *trnE_uuc* | *trnF_gaa* | *trnfM_cau* | *trnG_gcc* | *trnG_ucc** | *trnH_gug* |
|  | *trnI_cau* x 2 | *trnI_gau** x 2 | *trnK_uuu** | *trnL_caa* x 2 | *trnL_uaa** | *trnL_uag* | *trnM_cau* | *trnN_guu* x 3 | *trnP_ugg* |
|  | *trnQ_uug* | *trnR_acg* x 2 | *trnR_ucu* | *trnS_gcu* | *trnS_gga* | *trnS_uga* | *trnT_ggu* | *trnV_gac* x 2 | *trnV_uac** |
|  | *trnW_cca* | *trnY_gua* |  |  |  |  |  |  |  |
| Pseudo gene | *ψ-clpP* | *ψ-rpl23* x 4 | *ψ-ndhE* |  |  |  |  |  |  |

*intron ^α^cp genome contains a copy of *rps12* exon 1 in LSC and two copies of *rps12* exon 2 and 3 in IR. ^β^*trnK* intron coded maturase. ^γ^TIG protein gene. x 2 indicates two copies of the gene.
