# Supplementary material for: accD nuclear transfer of Platycodon grandiflorum and the plastid of early Campanulaceae
Source: BMC Genomics. 2017 Aug 11;18:607. doi: 10.1186/s12864-017-4014-x (PMC5553655; doi:10.1186/s12864-017-4014-x)
Supplement: Supplementary file 2 — Cytidine (C) to uridine (U) editing sites in the chloroplast genome of Platycodon validated by RNA-Seq data from leaf, root, stem, seed, petal, pistil, sepal, and stamen. (DOCX 21 kb) [file 12864_2017_4014_MOESM2_ESM.docx]

Table S2

| No. | Region | Gene | Strand | Total | Site (bp) | Leaf | Root | Stem | Seed | Petal | Pistil | Sepal | Stamen |
| --- | --- | --- | --- | --- | --- | --- | --- | --- | --- | --- | --- | --- | --- |
| 1 | LSC | *rps14* | - | 1 | 12,401 | G → A | - | G → A | G → A | G → A | - | G → A | - |
| 2 |  | *rps18* | - | 1 | 26,803 | G → A | G → A | - | G → A | G → A | G → A | G → A | - |
| 3 |  | *petL* | - | 1 | 29,255 | G → A | G → A | G → A | G → A | G → A | G → A | G → A | - |
| 4 |  | *ndhK* | - | 1 | 48,595 | G → A | - | G → A | - | G → A | G → A | G → A | - |
| 5 |  | *rps2* | - | 1 | 53,587 | G → A | G → A | - | G → A | G → A | G → A | G → A | G → A |
| 6 |  | *atpA* | - | 1 | 68,037 | G → A | - | G → A | - | G → A | G → A | G → A | - |
| 7 |  | *atpF* | - | 1 | 70,070 | G → A | G → A | - | - | G → A | G → A | G → A | - |
| 8 |  | *petB* | + | 2 | 75,722 | C → T | - | C → T | - | C → T | C → T | C → T | C → T |
|  |  |  |  |  | 75,915 | C → T | - | C → T | C → T | C → T | C → T | C → T | C → T |
| 9 |  | *rpoA* | - | 1 | 78,346 | G → A | G → A | - | G → A | - | G → A | G → A | - |
| 10 | IRA | *ndhB* | - | 3 | 96,119 | G → A | G → A | G → A | - | - | G → A | G → A | - |
|  |  |  |  |  | 96,238 | G → A | G → A | G → A | G → A | G → A | G → A | G → A | - |
|  |  |  |  |  | 96,556 | G → A | - | G → A | - | G → A | G → A | G → A | - |
| 11 |  | *ndhH* | + | 1 | 116,860 | C → T | C → T | - | C → T | - | C → T | C → T | - |
| 12 |  | *ndhA* | + | 2 | 119,509 | C → T | C → T | C → T | - | C → T | C → T | C → T | - |
|  |  |  |  |  | 119,621 | C → T | C → T | C → T | C → T | C → T | C → T | C → T | - |
| 13 |  | *ndhG* | + | 1 | 120,987 | C → T | C → T | C → T | - | C → T | C → T | C → T | - |
| 14 | SSC | *ndhD* | - | 1 | 128,239 | G → A | G → A | G → A | - | - | - | - | - |
| 15 | IRB | *ndhG* | - | 1 | 129,944 | G → A | G → A | G → A | - | G → A | G → A | G → A | - |
| 16 |  | *ndhA* | - | 2 | 131,310 | G → A | G → A | G → A | G → A | G → A | G → A | G → A | - |
|  |  |  |  |  | 131,422 | G → A | G → A | G → A | - | G → A | G → A | G → A | - |
| 17 |  | *ndhH* | - | 1 | 134,071 | G → A | G → A | - | G → A | - | G → A | G → A | - |
| 18 |  | *ndhB* | + | 3 | 154,375 | C → T | - | C → T | - | C → T | C → T | C → T | - |
|  |  |  |  |  | 154,693 | C → T | C → T | C → T | C → T | C → T | C → T | C → T | - |
|  |  |  |  |  | 154,812 | C → T | C → T | C → T | - | - | C → T | C → T | - |

*-, No alignment
