# Supplementary material for: accD nuclear transfer of Platycodon grandiflorum and the plastid of early Campanulaceae
Source: BMC Genomics. 2017 Aug 11;18:607. doi: 10.1186/s12864-017-4014-x (PMC5553655; doi:10.1186/s12864-017-4014-x)
Supplement: Supplementary file 3 — Plastid tRNA and rRNA gene distribution among Campanulaceae species. ‘+’ indicates presence of the gene, and ‘-’ indicates complete absence of the gene. ‘ΨΨ’ indicates two pseudo-copies of the gene. The number of ‘+’ indicates the copy number of the gene. (DOCX 16 kb) [file 12864_2017_4014_MOESM3_ESM.docx]

|  | Platycodon | Hanabusaya | Trachelium | Campanula | Adenophora |  | Platycodon | Hanabusaya | Trachelium | Campanula | Adenophora |
| --- | --- | --- | --- | --- | --- | --- | --- | --- | --- | --- | --- |
| *trnA_ugc* | + + | + + | + + | + + | + + | *trnP_ugg* | + | + | + | + | + |
| *trnC_gca* | + | + | + | + | + | *trnQ_uug* | + | + | + | + | + |
| *trnD_guc* | + | + | + | + | + | *trnR_acg* | + + | + + | + + | + + | + + |
| *trnE_uuc* | + | + | + | + | + | *trnR_ucu* | + | + | + | + | + |
| *trnF_gaa* | + | + | + | + | + | *trnS_gcu* | + | + | + | + | + |
| *trnfM_cau* | + | + | + | + | + | *trnS_gga* | + | + | + | + | + |
| *trnG_gcc* | + | + | + | + | + | *trnS_uga* | + | + | + | + | + |
| *trnG_ucc* | + | + | + | + | + | *trnT_ggu* | + | — | + | — | — |
| *trnH_gug* | + | + | + | + | + | *trnT_ugu* | — | + | + | + | + |
| *trnI_cau* | + + | + + | + + | + + | + + | *trnV_gac* | + + | + + | + | + | + + |
| *trnI_gau* | + + | + + | + + | + + | + + | *trnV_uac* | + | + | + | + | + |
| *trnK_uuu* | + | + | + | + | + | *trnW_cca* | + | + | + | + | + |
| *trnL_caa* | + + | + + | + + | + + | + + | *trnY_gua* | + | + | + | + | + |
| *trnL_uaa* | + | + | + | + | + | 23S *rRNA* | + + | + + + + | + + + + | + + | + + |
| *trnL_uag* | + | + | + | + | + | 16S *rRNA* | + + | + + | + + | + + | + + |
| *trnM_cau* | + | + | + | + | + | 5S *rRNA* | + + | + + | + + | + + | + + |
| *trnN_guu* | + + + | + + | + + | + + | + + | 4.5S *rRNA* | + + | + + | + + | + + | + + |

Table S3
