# Supplementary material for: accD nuclear transfer of Platycodon grandiflorum and the plastid of early Campanulaceae
Source: BMC Genomics. 2017 Aug 11;18:607. doi: 10.1186/s12864-017-4014-x (PMC5553655; doi:10.1186/s12864-017-4014-x)
Supplement: Supplementary file 4 — Plastid intron distribution in Campanulaceae species and other related taxa. ‘+’ indicates presence of the gene, ‘Ψ’ marks pseudo-copy of the gene, and ‘-’ indicates complete absence of the gene. ‘ΨΨ’ indicates two pseudo-copies of the gene. (DOCX 18 kb) [file 12864_2017_4014_MOESM4_ESM.docx]

|  | Asterales | | | | | | | Apiales | | | |
| --- | --- | --- | --- | --- | --- | --- | --- | --- | --- | --- | --- |
|  | Campanulaceae | | | | | Asteraceae | | Apiaceae | | Araliaceae | |
|  | Platycodon | Hanabusaya | Trachelium | Campanula | Adenophora | Halianthus | Lactuca | Daucus | Anthriscus | Panax | Eleutherococcus |
| **GroupⅠ** |  |  |  |  |  |  |  |  |  |  |  |
| *trnL_uaa*.i | + | + | + | + | + | + | + | + | + | + | + |
| **Group Ⅱ** |  |  |  |  |  |  |  |  |  |  |  |
| *atpF*.i | + | + | + | + | + | + | + | + | + | + | + |
| *clpP*.i1 | - | - | ΨΨ | ΨΨ | ΨΨ | + | + | + | + | + | + |
| *clpP*.i2 | Ψ | ΨΨ | ΨΨ | ΨΨ | ΨΨ | + | + | + | + | + | + |
| *ndhA*.i | + + | + + | + + | + + | + + | + | + | + | + | + | + |
| *ndhB*.i | + + | + | + | + | Ψ | + + | + + | + + | + + | + + | + + |
| *petB*.i | + | + | + | + | + | + | + | + | + | + | + |
| *petD*.i | + | + | + | + | + | + | + | + | + | + | + |
| *rpl16*.i | + + | + | + | + | + | + | + | + | + | + | + |
| *rpl2*.i | + + | + | + | + | + | + | + | + | + | + | + |
| *rpoC1*.i | - | + | + | + | + | + | + | + | + | + | + |
| *rps12*.i1t1 | + + | + | + | + | + | + + | + + | + + | + + | + + | + + |
| *rps12*.i1t2 | + | + + | + + | + + | + + | + | + | + | + | + | + |
| *rps12*.i2 | + | + + | + + | + + | + + | + | + | + | + | + | + |
| *rps16*.i | - | - | - | - | - | + | + | + | + | + | + |
| *ycf3*.i1 | + | + | + | + | + | + | + | + | + | + | + |
| *ycf3*.i2 | + | + | + | + | + | + | + | + | + | + | + |
| *trnA_ugc*.i | + + | + + | + + | + + | + + | + + | + + | + + | + + | + + | + + |
| *trnG_ucc*.i | + | + | + | + | + | + | + | + | + | + | + |
| *trnI_gau*.i | + + | + + | + + | + + | + + | + + | + + | + + | + + | + + | + + |
| *trnK_uuu*.i | + | + | + | + | + | + | + | + | + | + | + |
| *trnV_uac*.i | + | + | + | + | + | + | + | + | + | + | + |

Table S4
