# Supplementary figures and images for: accD nuclear transfer of Platycodon grandiflorum and the plastid of early Campanulaceae
Source: BMC Genomics. 2017 Aug 11;18:607. doi: 10.1186/s12864-017-4014-x (PMC5553655; doi:10.1186/s12864-017-4014-x)

## Slide 1
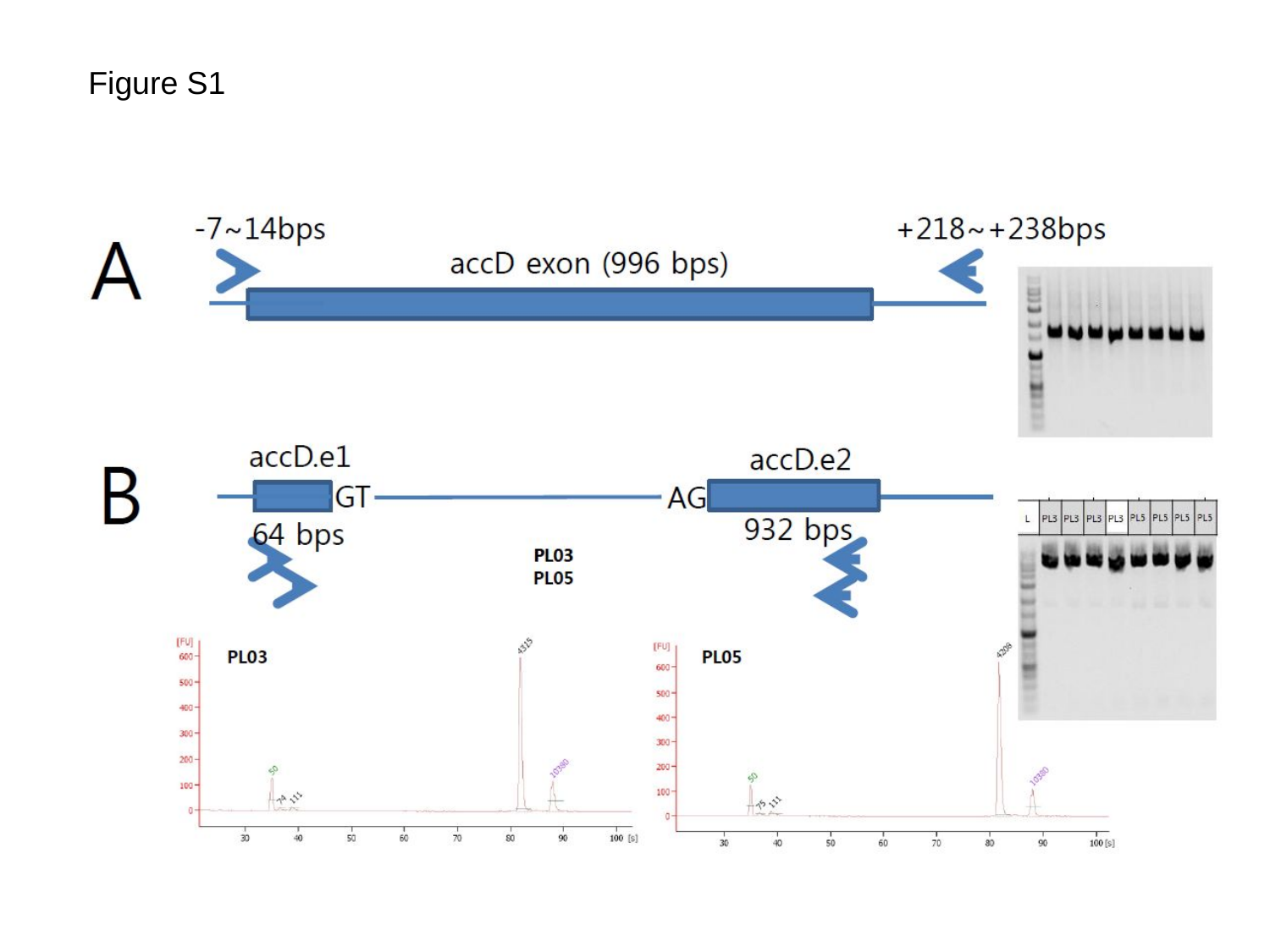

Figure S1

Supplement: Supplementary file 5 — cDNA and DNA PCR confirmation of nr-accD in Platycodon grandiflorum. A: The size of the nr-accD cDNA sequence, primer sites, and cDNA PCR products in P. grandiflorum cultivars. B: The size of the nr-accD DNA sequence, primer sites, and genomic DNA PCR products in P. grandiflorum cultivars. PL03 [accD_PL_LPRF ~ accD_PL_LPR] and PL05 [accD_PL_LPRF1 ~ accD_PL_LPR1]. (PPTX 757 kb) [file 12864_2017_4014_MOESM5_ESM.pptx]

## Slide 1
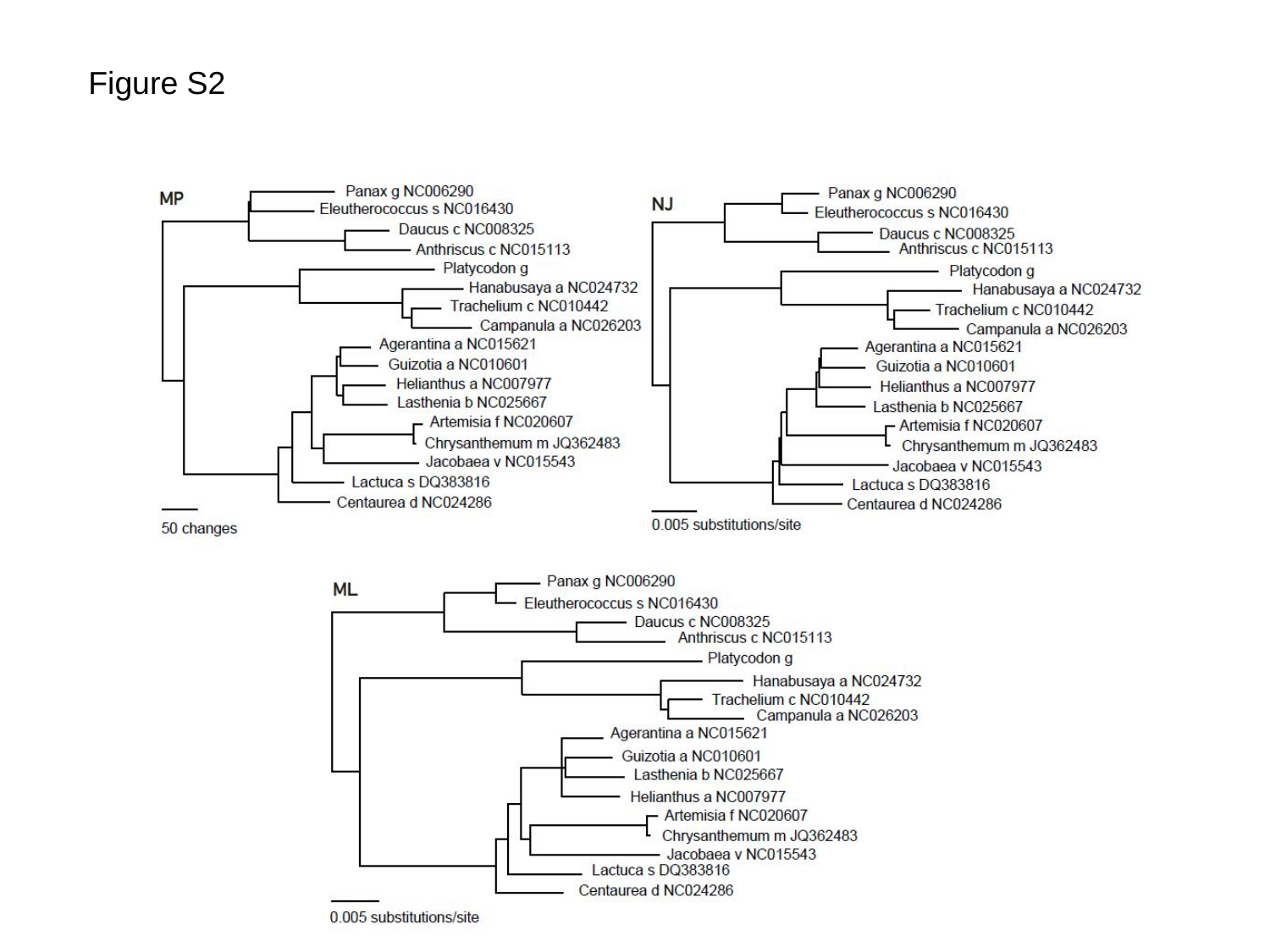

Figure S2

Supplement: Supplementary file 6 — Phylogenetic trees generated from the DNA sequences of seven cp-genes using three algorithms. (A) single maximum parsimonious (MP) tree, (B) single neighbor-joining (NJ) tree, and (C) single maximum likelihood (ML) tree (HYK85 + G + I model). (PPTX 749 kb) [file 12864_2017_4014_MOESM6_ESM.pptx]

## Slide 1
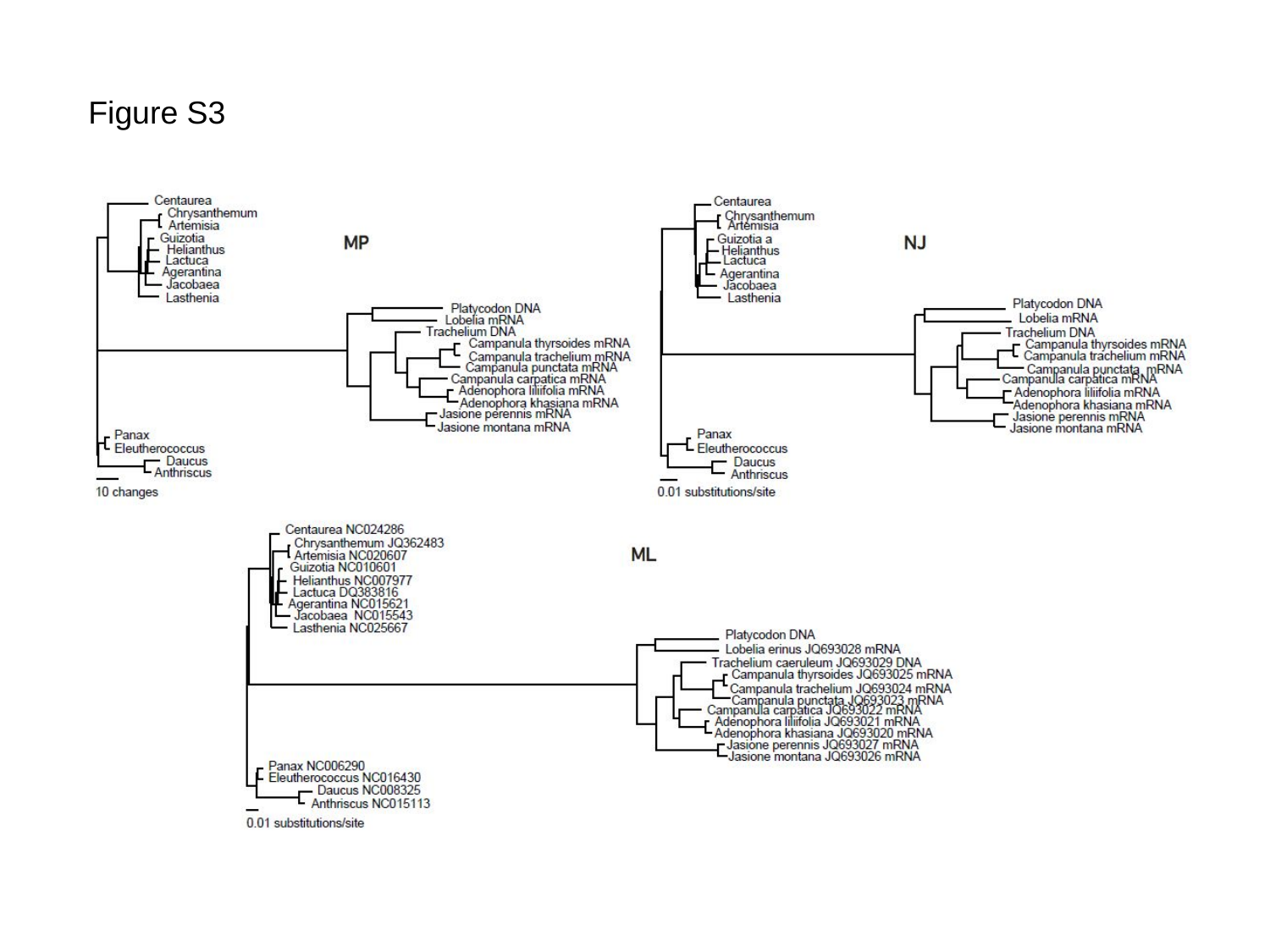

Figure S3

Supplement: Supplementary file 7 — Phylogenetic trees generated from the DNA sequences of the accD gene using three algorithms. (A) single maximum parsimonious (MP) tree, (B) single neighbor-joining (NJ) tree, and (C) single maximum likelihood (ML) tree (HYK85 + G + I model). (PPTX 857 kb) [file 12864_2017_4014_MOESM7_ESM.pptx]
